# Recurrence Quantification Analysis of Heart Rate During Mental Arithmetic Stress in Young Females

**DOI:** 10.3389/fphys.2020.00040

**Published:** 2020-02-11

**Authors:** Dimitriy Dimitriev, Elena V. Saperova, Aleksey Dimitriev, Yuriy Karpenko

**Affiliations:** ^1^Department of Biology, Chuvash State Pedagogical University named I Ya Yakovlev, Cheboksary, Russia; ^2^Centre for Strategic Planning, Russian Ministry of Health, Moscow, Russia

**Keywords:** mental arithmetic stress, heart rate variability, non-linear analysis, recurrence plot, recurrence quantification analysis

## Introduction

The heart rate regulation system was conceptualized as a complex network, with non-linear feedforward and feedback inputs. This system exhibits chaotic and non-linear dynamics, due to interactions between physiological oscillators, functional state changes, and noise (Voss et al., 2008).

Recurrence is a common feature of dynamical systems. The recurrence plot (RP) displays specific large- and small-scale patterns, which are produced by typical dynamical behavior (Eckmann et al., 1995), e.g., diagonals (similar local time evolution of different parts of the trajectory), or vertical and horizontal black lines (the state does not change for some time). Recurrence plot is suitable for analysis of short, noisy and non-stationary sequences of RR intervals and RP is sensitive to small changes in the system's dynamics (Javorka et al., 2008).

Recurrence quantification analysis (RQA) is a useful toolkit for studying the dynamics of complex systems, such as laminar, divergent, or non-linear transition behaviors (Marwan et al., 2002). Short line segments parallel to the main diagonal are essential features of an RP, indicating that the evolution of states is similar at different times, and that the process could be deterministic. If these diagonal line structures occur beside single isolated points, the process could be chaotic. The length of such diagonal line structures relies on the predictability and the dynamics of the system (periodic, chaotic, or stochastic) (Webber, 2007; Marwan et al., 2009). The stability of one state's state causes vertical lines in the RP. Theoretically, diagonal and vertical linear structures are inherent to the deterministic process, but not for the random process. RQA was proposed for the analysis of non-linear dynamical systems, by means of quantifying the diagonal and vertical lines of RP (Marwan et al., 2002). High values of RQA measures trapping time (TT); laminarity (LAM), mean length of a diagonal line (LMEAN), and maximum length of a diagonal line (LMAX) imply low complexity in the system's dynamics; and LMAX is negatively correlated with a Large Lyapunov Exponent (LLE), which is a key indicator of chaos (Eckmann et al., 1995). RQA is sufficiently sensitive for assessment of changes in sympathetic and parasympathetic activity, induced by active orthostatism and pharmacological interventions (Mestivier et al., 1998; Javorka et al., 2008). RQA indicators have been used for the detection of real-life stress and emotion recognition from multimodal data (Torres-Valencia et al., 2017).

Mental arithmetic (MA) (e.g., successive subtraction of a simple number from a large number) is known as one of the substantial tasks that reliably impacts on heart rate variability. MA induces workload, which could lead to cognitive overload, increasing blood pressure, and a reduction in vagal activity (Hunt et al., 2017; Chin et al., 2018). The mental arousal that follows MA produces a significant decrease in symbolic dynamics parameters, and changes in time reversibility of RR intervals (Visnovcova et al., 2014). Repeated mental workload during a high-paced video game has been associated with a significant reduction in RQA indicators of heart rate variability (HRV) dynamics (Castaldo et al., 2017).

We carried out an RQA of ECG records obtained during rest, and during MA stress. The comparison of RQA parameters for both states potentially enable identification of RQA parameters that are sensitive to MA stress. With this approach, we aimed to provide a dataset for a better understanding of non-linear behavior of heart rate during MA stress and for an assessment of changes in recurrence parameters of heart rate associated with mental workload.

## Materials and Methods

Fifty healthy non-smoking female students (21.1 ± 1.9 years) participated in this study. We asked all participants to refrain from vigorous physical activity, alcohol, and caffeine for 12 h prior to the experiment.

The study was approved by the local ethics committee, and informed consent was obtained from each participant.

Participants performed MA for 10 min, by continuously subtracting 7 from a 3-digit number. An ECG signal was recorded with a standard lead-II setup (Poly-Spectrum-8/E, Neurosoft Inc, 2000 Hz sampling frequency, 0.05–0.75 Hz bandpass filter, drift filter 0.5, 50 Hz notch filter) for 10 min twice—before mental stress and during MA, in the supine position. R-peak detection and RR preprocessing (artifact correction and detrending) within ECG were conducted using Kubios HRV premium software (Tarvainen et al., 2014).

State space reconstruction of RR data was performed, based on the standard delay embedding method. To determine the correct embedding dimension, Cao's method (Cao, 1997) was used. Calculation of E(1) yielded saturation values at rest ranging from 7 to 11 (mean and standard error 9.58 ± 0.14). In accordance with previous studies, we used m = 10 for the phase space reconstruction (Dabiré et al., 1998; González et al., 2014). The time delays (τ) for the RPs were calculated as the first minimum of the average mutual information function. We constructed a square matrix of Euclidean distances between phase space points (i.e., between states of the system at a given time). The tolerance level was selected separately for each recording.

In order to construct RP, we compute the recurrence matrix for reconstructed states **x**_i_ and **x**_*j*_

$$\begin{array}{l} R_{i,j}\left(\varepsilon \right)=\theta \left\{\varepsilon -{\text{x}}_{i}-{\text{x}}_{j}\right\}, \end{array}$$

with θ {·} being the Heaviside function, and ε is an arbitrary threshold.

For the analysis of the RR time series, we used the RQA parameters: recurrence rate (REC), determinism (DET), LMAX, LMEAN, LAM, maximal vertical length (Vmax), TT, and Shannon entropy (ShanEn) (Martínez et al., 2017).

The REC is the density of the RP on the phase space trajectory, or the ratio of ones and zeros in the RP matrix, as follows:

$$\begin{array}{l} REC=\frac{1}{N^{2}}\overset{N}{\underset{i,j=1}{\sum }}R_{i,j} \end{array}$$

DET is an indicator of the regularity and determinism of the system dynamics. DET is computed as a percentage of recurrence points on the diagonal lines, as follows:

$$\begin{array}{l} DET=\frac{\sum_{l=l_{min}}^{N}lP\left(l\right)}{\sum_{i,j}^{N}R_{i,j}}, \end{array}$$

where P(l) is the distribution of diagonal l line lengths.

Marwan et al. (2009) defined the LAM of an RP as a fraction of recurrence points that form vertical lines, as follows:

$$\begin{array}{l} LAM=\frac{\sum_{v=v_{min}}^{N}vP\left(v\right)}{\sum_{v=1}^{N}vP\left(v\right)}, \end{array}$$

where P(v) is the distribution of the length of vertical lines. LAM quantifies the occurrence of laminar states in the system.

The TT represents the length of time that the dynamics remain trapped in a certain state. TT is the average length of vertical lines in the RP, as follows:

$$\begin{array}{l} TT=\frac{\sum_{v=v_{min}}^{N}vP\left(v\right)}{\sum_{v=v_{min}}^{N}\;P\left(v\right)}. \end{array}$$

The ShanEn of the line length distribution is defined as follows:

$$\begin{array}{l} ShanEn=\overset{l}{\underset{l=l_{min}}{\sum }}n_{l}\ln\;n_{l}, \end{array}$$

where *n*_*l*_ is the ratio of lines *l* to the number of all lines.

The LMAX is inversely related to the most positive Lyapunov exponent, i.e., high LMAX indicates that the system is less chaotic. The Vmax is the maximum of all durations of the laminar states.

To determine threshold distance ε, we calculated REC for 11 different values of ε (from 0.5 to 10% of maximum phase diameter). Similarly, we calculated DET and LAM for nine values of a minimum line (from 2 to 10) in order to define minimum line lengths for diagonal (DET) and vertical (LAM) structures. We used the embedded MATLAB function “rankfeatures” (with “CriterionValue” set to “roc”) to obtain a value for each scale that would optimize discrimination between rest and mental stress (Almeida et al., 2018).

The results of rankfeatures calculation indicate that 4% of maximal space diameter provided the best discrimination between rest and stress (Figure S1A, Table S1). Similarly, results for DET and LAM suggest that a minimum line length of 2 should be considered for calculation of DET, LAM, TT, and ShanEn (Figures S1B,C, Table S2).

In addition, spectral powers (LF and HF), the standard deviation of all RR intervals (SDNN), short-term scaling exponent (α1) and long-term scaling exponent (α2) of DFA were calculated for each sequence of RR intervals.

To classify RR sequences, we explored linear discriminant analysis (LDA) commonly used for detection of stress (Melillo et al., 2011; Kaur et al., 2014). We used a log-transformation to meet assumptions of homogeneity of variance and normally-distributed residuals. The homogeneity of variances was tested by means of Cochran C statistic. In order to evaluate the overall performance of the classifiers, results of the rest-stress comparisons were quantified by the criteria of sensitivity, specificity, precision, and accuracy.

Because all variables were not normally distributed, we tested the null hypothesis that RQA measures were the same for rest and stress by means of the Wilcoxon matched pair test. Testing was performed using a significance level *p* = 0.05. Values of RQA measures are expressed as the mean ± standard error of the mean (SE).

## Recurrence Plot, RQA, and HRV Measures During Rest and MA Stress

**Figure 1:**
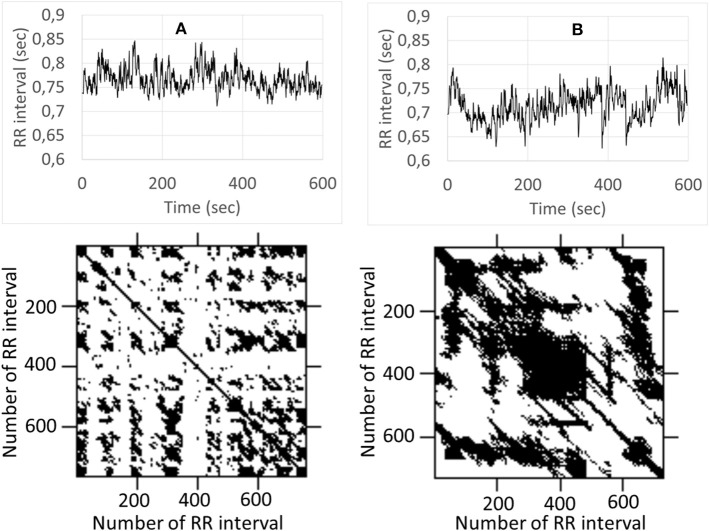
Examples of the RP and RR for rest **(A)** and mental stress **(B)**. Upper parts of **(A,B)** depict variation of R-R intervals, the lower graphs demonstrate corresponding recurrence plot.

As an initial step to analyze the recurrence of heart rate, we drew an RP (Figure 1). The RP showed a consistent pattern of more clustering of points during mental stress, with respect to the rest (Figure 1). This suggests that recurrence of heart rate underwent a notable evolution during the transition from rest to mental stress. Thus, the RPs can sensitively reflect the signals from different physiological states. The RPs during mental stress are characterized by longer vertical lines (TT) and a higher percentage of points forming diagonal lines (DET). The results of the research indicate that a deterministic structure was present in the heart rate dynamics (DET was statistically >0), but that heart rate variations were not completely deterministic (DET was statistically <1). The statistical analysis of RQA measures reveals significant changes in REC, DET, LAM, LMAX, Vmax, and TT during mental stress (Table 1). MA induced a significant increase of DET to rather a high level. A high level of DET associated with a high predictability of the heart rate regulation system (Marwan et al., 2009). Mental stress elicits a significant increase in the maximum length of the diagonal line LMAX (and decrease in LLE), which indicates that the sensitivity of the heart rate regulation system is diminished to the initial conditions (Eckmann et al., 1995). We compared rest and stress levels of LAM and TT, and found the long permanence of the system in a particular state during mental stress. Heart rate RQA was recently used for discrimination between young and elderly subjects (Singh et al., 2019).

**Table 1:** RQA indexes analysis during rest and mental stress.

	**REST**	**MENTAL STRESS**	***p***
REC	0.03 ± 0.003	0.05 ± 0.007	<0.05
DET	0.41 ± 0.02	0.51 ± 0.03	<0.001
LAM	0.46 ± 0.03	0.62 ± 0.03	<0.001
LMAX	83.56 ± 15.92	212.78 ± 37.44	<0.001
LMEAN	6.16 ± 0.70	4.72 ± 0.56	>0.05
Vmax	20.50 ± 4.00	30.52 ± 4.42	<0.01
TT	2.67 ± 0.10	3.26 ± 0.18	<0.001
ShanEn	1.22 ± 0.14	1.25 ± 0.11	>0.05

*Data are reported as the mean ± standard error*.

The HF component of HRV was significantly reduced in the mental stress (576.11 ±107.18 ms^2^) compared with the rest (1143.92 ± 198.27 ms^2^) (*P* < 0.01) and the LF/HF ratio was significantly higher in the mental stress session (1.84 ± 0.16) compared with the rest (1.31 ± 0.14) (*P* < 0.01). There was no difference in the LF component of HRV between the mental stress (686.82 ± 118.62 ms^2^) and the rest (920.77 ± 135.3 ms^2^) (*P* > 0.05). MA induced significant decrease in SDNN (from 43.69 ± 3.07 ms to 34.42 ± 2.56 ms, *P* < 0.01). Short-term scale of DFA α1 was significantly higher during mental stress than in rest period (1.17 ± 0.03 vs. 1.0 ± 0.03, *p* < 0.01). Mental stress influenced long-term fractal properties of heart rate fluctuation: α2 showed similar increase from 0.35 ± 0.02 during rest to 0.43 ± 0.01 in mental stress session (*P* < 0.05).

The highest estimates of the total classification accuracy, sensitivity, and specificity was achieved by α1 and by recurrent plot measures (Table S3).

The data presented in our database may be sufficient for detection of a cognitive workload in performing MA.

## Data Availability Statement

The datasets generated for this study can be found in the Figshare data repository https://figshare.com/articles/The_effect_of_mental_arithmetic_stress_on_the_nonlinear_dynamics_of_heart_rate_in_young_females_recurrence_quantification_analysis_/8345678.

## Ethics Statement

The studies involving human participants were reviewed and approved by Ethical Committee for biomedical research of Chuvash State University named I. N. Ulyanov. The patients/participants provided their written informed consent to participate in this study. Written informed consent was obtained from the individual(s) for the publication of any potentially identifiable images or data included in this article.

## Author Contributions

DD, ES, AD, and YK designed the study approach and experiment. DD and ES collected the research material. DD, AD, and YK were responsible for analysis of the data. DD wrote the manuscript with contributions from ES, AD, and YK. All authors read and approved the final manuscript.

### Conflict of Interest

The authors declare that the research was conducted in the absence of any commercial or financial relationships that could be construed as a potential conflict of interest.
