# Supplementary material for: Recurrence Quantification Analysis of Heart Rate During Mental Arithmetic Stress in Young Females
Source: Front Physiol. 2020 Feb 11;11:40. doi: 10.3389/fphys.2020.00040 (PMC7026015; doi:10.3389/fphys.2020.00040)
Supplement: Supplementary file 1 [file Data_Sheet_1.PDF]

Recurrence quantification analysis of heart rate during mental arithmetic stress in young females

Dimitriy Dimitriev, Elena V. Saperova, Aleksey Dimitriev, Yuriy Karpenko·

Supplement

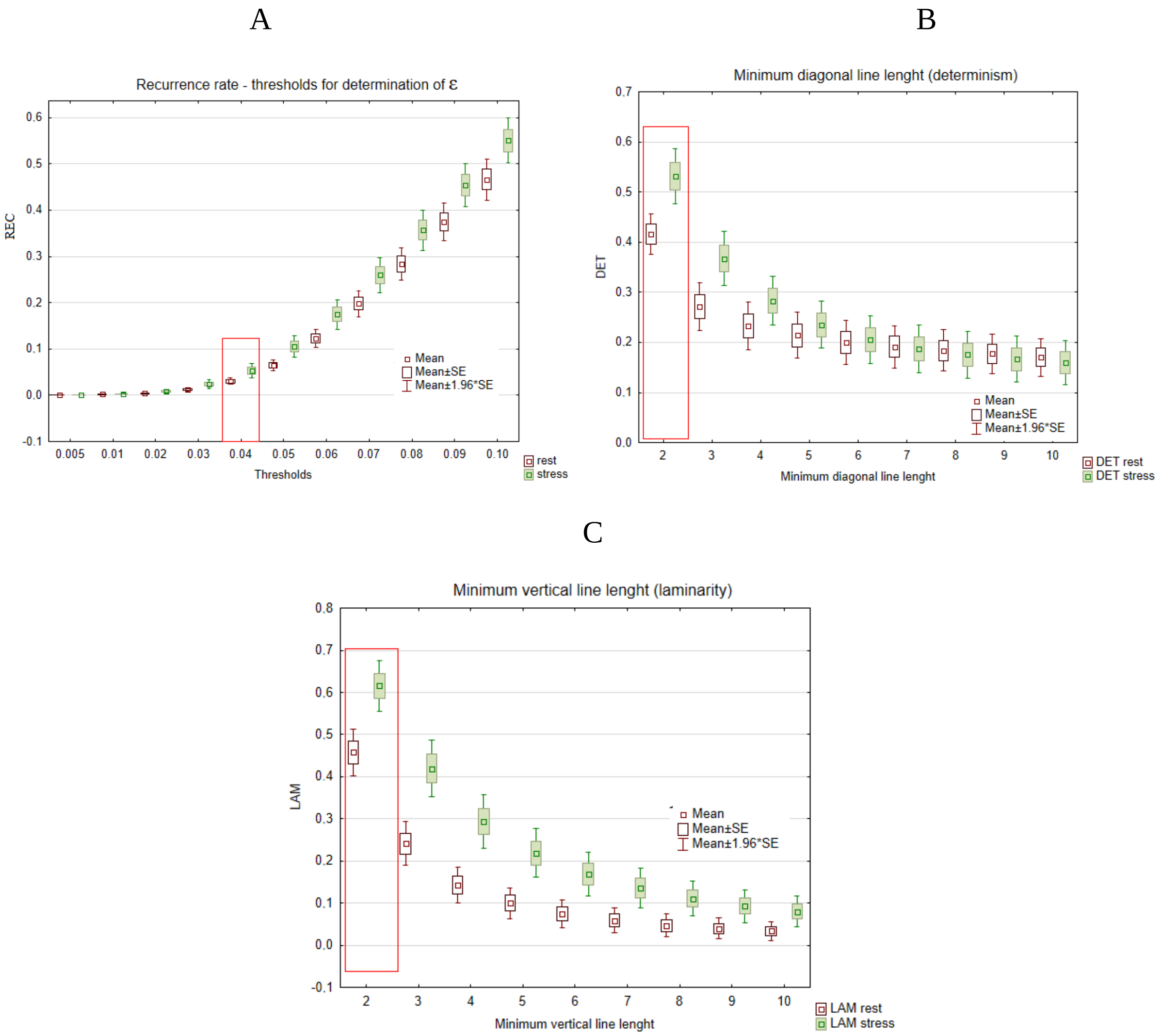

Figure S1. The determination of *ε* (A) and the minimum line length for the calculation of DET (B) and LAM (C).

Table S1.
The area under the ROC (AUROC) curves for the discrimination between rest and mental stress considering RR as the discriminator. RR was calculated for eleven different values of ε, varying from 0.5% to 10% of the maximum phase space distance. The marked value indicates the chosen threshold for ε.

% of max phase space distance	0.5%	1%	2%	3%	4%	5%	6%	7%	8%	9%	10%
AUROC	0.158	0.0092	0.181	0.1727	<u>0.2079</u>	0.1688	0.1458	0.1576	0.1515	0.1519	0.1719

Table S2.
The AUROC for the discrimination between rest and mental stress considering DET and LAM as the discriminators, calculated with different minimum line lengths. The marked values indicate the values chosen for DET and LAM.

Min line length	2	3	4	5	6	7	8	9	10
DET	0.1988	0.1628	0.1224	0.0781	0.0247	0.0043	0.0234	0.0386	0.0425
LAM	0.2331	0.2318	0.2166	0.2005	0.1871	0.1910	0.1858	0.1758	0.1801

Table S3.

Performance of the classification rules based on single HRV parameters

	**ACC**	**SEN**	**SPE**	**PPV**	**NPV**
**SDNN**	0.59	0.62	0.56	0.58	0.60
**LF**	0.54	0.56	0.52	0.54	0.54
**HF**	0.59	0.58	0.60	0.59	0.59
**LF/HF**	0.65	0.62	0.68	0.66	0.64
**α1**	0.68	0.74	0.62	0.66	0.70
**α2**	0.63	0.64	0.62	0.63	0.63
**DET**	0.65	0.56	0.74	0.68	0.66
**LAM**	0.67	0.70	0.64	0.66	0.68
**LMAX**	0.61	0.42	0.80	0.68	0.58
**LMEAN**	0.54	0.68	0.37	0.55	0.53
**Vmax**	0.59	0.44	0.74	0.63	0.57
**TT**	0.62	0.46	0.78	0.68	0.59
**ShanEn**	0.52	0.24	0.70	0.53	0.51

ACC - Total classification accuracy
SEN -Sensitivity
SPE – Specificity
PPV - Positive Predictive Value
NPV - Negative Predictive Value
